# Biological insights and novel biomarker discovery through deep learning approaches in breast cancer histopathology

**DOI:** 10.1038/s41523-023-00518-1

**Published:** 2023-04-06

**Authors:** Divneet Mandair, Jorge S. Reis-Filho, Alan Ashworth

**Affiliations:** 1 UCSF Helen Diller Family Comprehensive Cancer Center, San Francisco, CA 94158 USA; 2 Memorial Sloan Kettering Cancer Center, New York, NY 10021 USA

**Keywords:** Cancer, Biomarkers

## Abstract

Breast cancer remains a highly prevalent disease with considerable inter- and intra-tumoral heterogeneity complicating prognostication and treatment decisions. The utilization and depth of genomic, transcriptomic and proteomic data for cancer has exploded over recent times and the addition of spatial context to this information, by understanding the correlating morphologic and spatial patterns of cells in tissue samples, has created an exciting frontier of research, histo-genomics. At the same time, deep learning (DL), a class of machine learning algorithms employing artificial neural networks, has rapidly progressed in the last decade with a confluence of technical developments - including the advent of modern graphic processing units (GPU), allowing efficient implementation of increasingly complex architectures at scale; advances in the theoretical and practical design of network architectures; and access to larger datasets for training - all leading to sweeping advances in image classification and object detection. In this review, we examine recent developments in the application of DL in breast cancer histology with particular emphasis of those producing biologic insights or novel biomarkers, spanning the extraction of genomic information to the use of stroma to predict cancer recurrence, with the aim of suggesting avenues for further advancing this exciting field.

## Introduction

Almost one-third of breast cancer cases recur in 10 years^1^ and decisions on who to treat aggressively early remain difficult. In the era of personalized medicine, histology, increasingly digitized and therefore more available for advanced computational methods, has been recognized as a significant resource of untapped information capable of improving our understanding of tumor biology and therapeutic efficacy at the individual patient level. The utilization and depth of genomic, transcriptomic and proteomic data for cancer has exploded over recent times and the addition of spatial context to this information, by understanding the correlating morphologic and spatial patterns of cells in tissue samples, has created an exciting frontier of research, histo-genomics^2^. At the same time, deep learning (DL), a class of machine learning algorithms employing artificial neural networks, has rapidly progressed in the last decade with a confluence of technical developments - including the advent of the modern graphic processing units (GPU), allowing efficient implementation of increasingly complex architectures at scale; advances in the theoretical and practical design of network architectures; and access to larger datasets for training - all leading to sweeping advances in image classification and object detection^3^. In the medical domain, DL has made great strides in the analysis of whole slide images (WSI), where both the limited amounts of available data and the large size of WSIs - often hundreds of thousands of pixels larger than those used typically in DL networks - had previously presented unique challenges in using these systems for predicting meaningful clinical and biologic outcomes. The confluence of these advances in digital pathology and artificial intelligence (AI) presents unique opportunities for prognostication, capturing molecular heterogeneity through new insights relating genomics to spatial content on histologic images and developing new biomarkers^4^ to guide diagnosis and therapy. Increasingly, academic institutions are adapting their data infrastructure to allow integration of such methods, with regulatory bodies such as the FDA advocating for AI-based methods to advance care^1^, with the first approval of an AI algorithm for cancer detection in pathology granted by the FDA in September 2021. Already, substantial progress has been made in developing systems that can reliably detect cancer, particularly in the context of the detection of prostate cancer in core biopsies and of breast cancer lymph node metastases^3^. In this review, we examine recent advances in the application of DL in breast cancer histology with particular emphasis on those producing biologic insights or novel biomarkers, spanning the extraction of genomic information to the use of stroma to predict cancer recurrence, with the aim of suggesting avenues for further advancing this exciting and potentially paradigm shifting field.

### A primer on DL

DL has been successful in applications dating back to the 1990s but only in recent years have the advances and acceptance of these techniques grown exponentially (Boxes 1 and 2). The field arose due to limitations in conventional machine learning techniques at processing data in raw form.

**Fig. 1:**
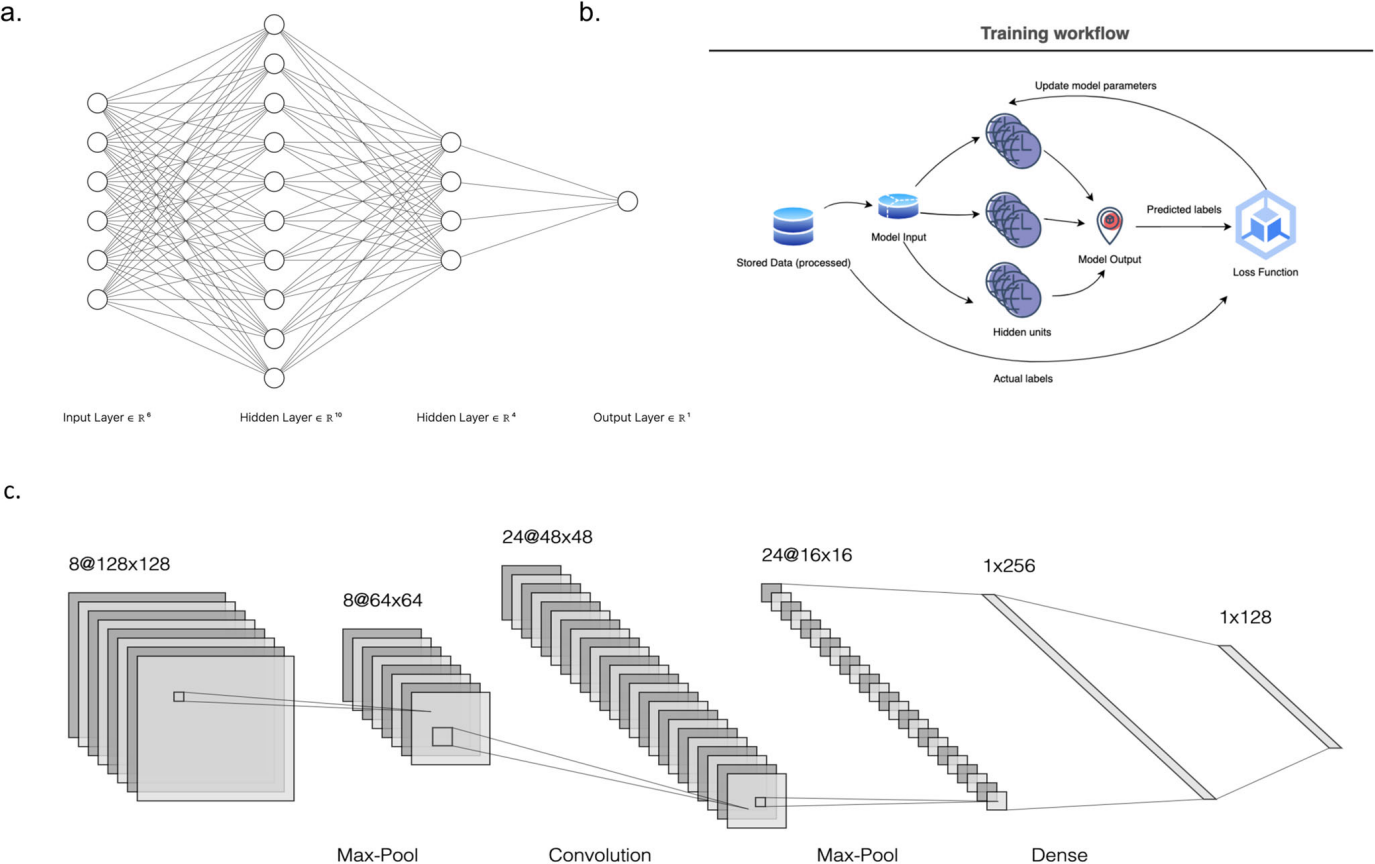
Example neural network architectures. **a** Basic neural network demonstrating input nodes with dimensionality of 8, two hidden layers that involve dimension expansion and subsequent reduction with a final output node. Each input node is connected to each of the hidden nodes (a fully connected network), with lines connecting nodes representing weights applied to a source to reach a destination. The above representation can also be visualized as a weight matrix, translating from the dimensionality of the input nodes to the dimensionality of the output nodes. **b** A pictorial the training process – inputs are fed to a DL network, predictions are made and compared to ground truth labels and parameters are updated in a loop. **c** A convolutional network architecture demonstrating the typical backbone of pooling layers followed by convolutional layers. Note that convolutions increase the number of filters while reducing dimensionality in the x/y dimensions (https://alexlenail.me/NN-SVG/).

Usually, features in a dataset would need to be hand-crafted or engineered to obtain optimal performance. DL, then, is a method of representation learning that allows a network (Fig. 1) to learn features from a dataset without such hand-crafted guidance^5^. DL networks, at a high level, work by feeding data through successive modules, each module consisting of mostly linear transformations with a non-linearity transformation added as a final step. While each individual module may be simple, as the number of modules or layers, increase, these networks can model quite complicated functions^5^.

**Fig. 2:**
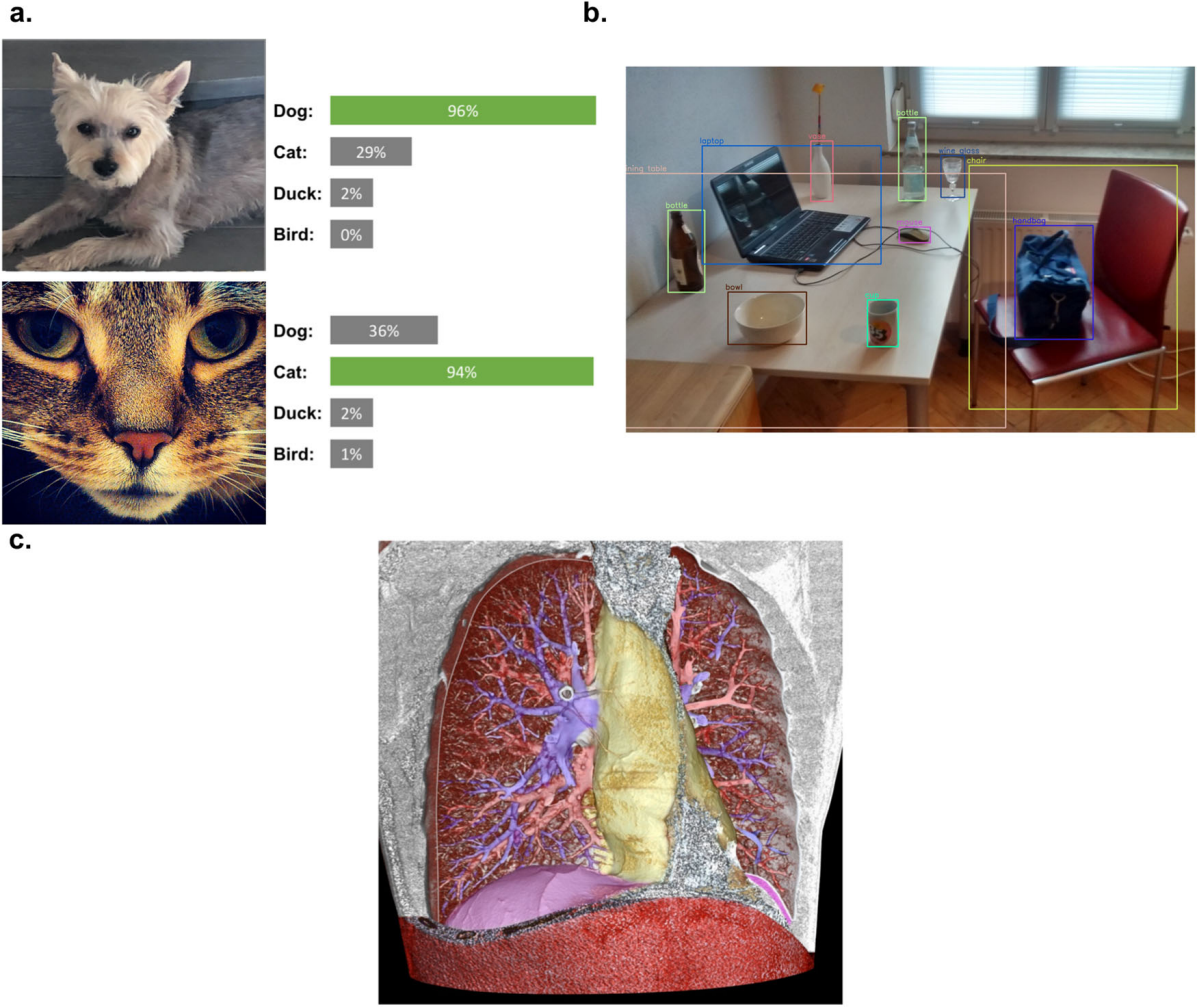
Image tasks in computer vision. Examples of (**a**) image classification, in which the task is one of classifying an image as one of 4 different types of animals (**b**) object detection, in which not only are objects classified but also identified in the image with boxes around their respective location and (**c**) image segmentation, where every pixel in the image is translated into some label, here the vessels, airways and contours of a lung. Sources: (**a**) the author’s photographs of dog (DM) and Jonesy the cat (AA). **b** https://commons.wikimedia.org/wiki/File:Detected-with-YOLO--Schreibtisch-mit-Objekten.jpg (**c**) https://commons.wikimedia.org/wiki/File:3D_CT_of_thorax.jpg.

As an example of how these networks are ‘trained’ to predict objects, we can imagine the task of predicting the type of animal in an image when our dataset consists of images of a variety of animals (Fig. 2). In deep learning, the neural network consists of multiple layers through which an image is successively transformed. Each image is fed through a network and for each category, or type of animal, the model outputs a score. Ideally, the model would predict the highest score for the correct category of animal in the image, an unlikely outcome if we use a randomly built model. To measure the degree to which the predicted category of the image aligns with the correct image category, a measure of error, or a loss function, is calculated^5^. The network can then modify its internal parameters, or weights, that produce the output of scores. This internal parameter update is done, parameter by parameter, through the calculation of gradients, which reflect how much the error rate in image classification changes when a given parameter is altered by a small amount. After such an internal update, the data are fed through the network again and the process is repeated. In this way, the representations learned from the image are done in an automatic manner that aligns with the task of interest^5^, in this case predicting the category of animal. In general, updates to the parameters of a network are performed over groups of training examples, or batches, as opposed to all the examples in a dataset at once, in a process called stochastic gradient descent. The prior example focuses on a classification task with qualitative labels as the output. Much of the same process would be replicated if the end outputs were not categorical, with the caveat that a new measure of error, or loss function, would be employed, with popular ones including the well-known mean-squared error. In this seemingly simple manner, exceptional results have been shown in areas from image processing to text and speech recognition^3^.

**Fig. 3:**
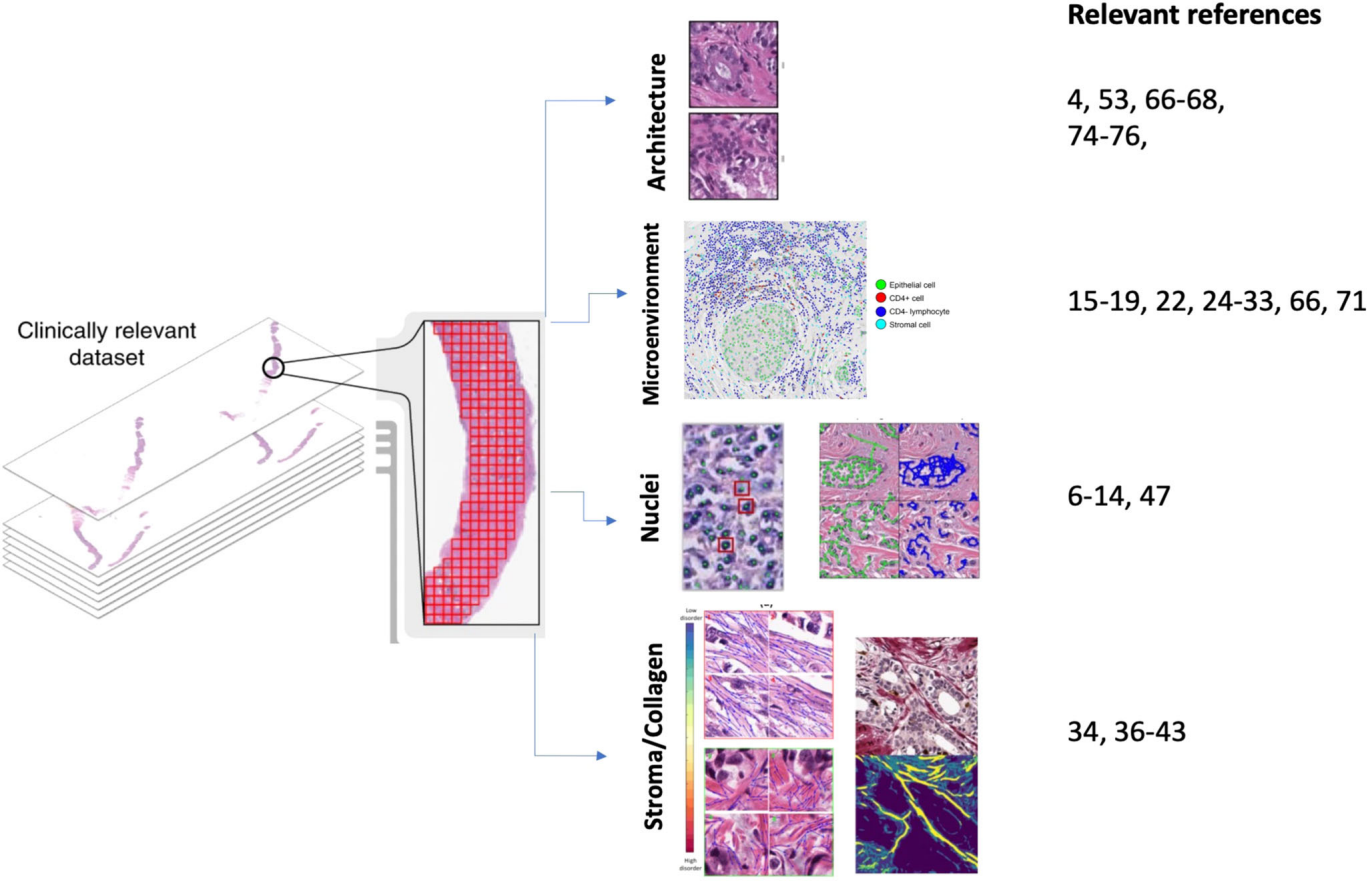
Deep learning features from WSI. Categories of features extracted from deep learning systems in breast cancer are illustrated, including invasive carcinoma architecture, cellular makeup of the microenvironment, nuclei features (including segmentation, orientation and nucleoli prominence), and stroma characteristics including collagen fiber orientation. On the right, references of studies exploring the detection of the different categories of features.

In the area of image detection, convolutional neural networks (CNN) form the initial backbone of networks that rival, and in some cases, surpass human level accuracy. These networks rely on two key aspects: convolutions and pooling. A convolutional layer, in the spirit of representation learning, applies a filter or kernel across an image input to produce a feature map. Numerous filters may be applied in a single layer, resulting in a stack of feature maps. Feature maps in an earlier part of a network correspond to vertical or horizontal edges in an image. Later maps may correspond to entire objects and even later maps show groupings of such objects^5^. The second aspect of these networks, pooling, allows the features detected to be average across an area of an image. In this way, the images detected are invariant to positioning. In this way, hierarchies of features in an image are built, with the overall system able to apply these learned features to detect images regardless of how they are positioned, rotated, or even how they appear^5^. Techniques for image prediction have continued to evolve (see Boxes 3 and 4), with the latest state of the art transformer networks, employing an attention mechanism across segments of an image, exceeding performance of traditional CNNs.

Box 1 definitions in AI In this review, classical machine learning refers to techniques, such as tree-based methods, support vector machine, etc. that require raw data to be first engineered into features and/or are in tabular (think excel) form. To clarify terminology, we define:

AI: theory and development of computer systems to perform tasks normally requiring ‘human’ intelligence.

Artificial General Intelligence: the theoretical (and far from any existing developments) development of an ‘agent’ capable of learning and performing any human level task.

Narrow AI: AI when applied in the context of a specific task, such as predicting the next word of a sentence or classifying an image.

Machine Learning: a subset of AI that includes techniques, applied to a specific task, which progressively improve performance or ‘learn’ when given increasing amounts of data.

Deep learning: A subset of machine learning that employs neural networks. Also characterized by readily handling extremely high dimensional, raw data input. For instance, a single RGB (color) image often contains (512 x 512 x 3) individual pixels. This raw form typically cannot be handled well by classical machine learning approaches but can be given to a neural network directly.

Box 2 learning with or without labels In the text, an example of supervised learning is discussed, where labels are known for existing data. Methods of learning include:

Supervised learning: This is where correct ‘labels’ for data (name of an object in an image, specific region of a histologic slide annotated by a pathologist, etc.) are known and these are generally filled out in a dataset by an expert. The machine learning system makes predictions and compares predicted labels to correct labels.

Strongly supervised learning: Every data sample has an expert that labels it. For instance, a WSI of a breast tissue is split into smaller pieces or tiles. A pathologist then marks each tile as containing cancer or not. A DL system emulates the pathologist by taking the tiles and learning to correctly predict cancer based on the pathologist’s label for each tile.

Weakly supervised learning: We now have genomic expression data for every patient. This data is not at the tile level – it corresponds to the entire WSI. We have a ‘label’ for a group of tiles but not all tiles might be reflective of this label (ie they might not all have morphology relevant to the expression patterns present in the sample). Yet we can still train a network by grouping together the tiles to predict the expression data. We might take a weighting of all the tiles in the WSI to make our prediction, or we might apply a more advanced ‘screen’ of the most likely-to-be-relevant group of tiles first. Regardless, weak supervision systems cluster inputs with a label and, if designed well, still make meaningful predictions.

Unsupervised learning: No labels are available for the data. Machine learning approaches in this category attempt to use the data itself (images, genomic sequences, etc.) to find consistent patterns, groupings or clustering implicit in the data. Examples include principal components, clustering methods, etc.

Self-supervised learning: A subset of unsupervised learning, self-supervised approaches also have no (or very minimal amounts) of labeled data available. These approaches, however, attempt train networks to perform the same tasks such as supervised techniques. A common approach to doing this is using contrastive techniques. A data sample is perturbed in some way (cropped, color is changed) and the two versions are fed to the network. The network is trained to group together images and their augmentations and separate different images. Doing so allows the network to learn useful representations of images without any labels. A variety of contrastive approaches (SimCLR, MOCO, Barlow Twin’s) have been developed. Once trained, if minimal amounts of labeled data are available, the networks can be ‘fine tuned’ with this data much like in classic supervised learning.

Box 3 image-related deep learning systems 3 common image-related tasks deep learning systems are trained to perform:

Image classification: Given an entire image, the network is asked to classify the image into one of several categories. A dog classifier, for instance, might classify a given image as belonging to one of 16 different dog breeds.

Object detection: The network is trained to not only recognize when a certain object is present (an animal) but point to where it is in an image (Fig. 2b). In an image of a living room, the network would output objects such as the sofa, TV and table, each with a box around the object in the image.

Image segmentation: The network is asked to classify every pixel in an image as belonging to one of a group of classes. Fig. 2c shows this for the vessels, airways and contours of a lung.

Box 4 attention and transformers Attention is a mechanism to weight different parts of an input and focus on the most relevant parts for the task at hand. To provide intuition, imagine an input is divided into smaller pieces – sentences are broken into tokens or images are broken into patches or tiles. Each token is then represented as 3 distinct vectors: a key, query and value. For a given token, we take its key vector and compare it against all the query vectors, performing a dot product with each. A dot product is higher if two vectors are ‘similar’ - thus this step amounts to weighting the other tiles in the image that are most similar to the tile we are currently looking at (our key). We then grab the corresponding value vectors, weighted by the prior dot products, giving us a final representation for our original token or tile. The key piece of a transformer architecture is the use of attention.

### Tumor characteristics

#### Cellular characteristics

##### Nuclei

Segmentation in digital pathology has extended beyond recognition of individual cell types and is capable of gleaning information at the level of nuclei that can be used to classify cells spatially more accurately or abstract second-order features that reflect a wealth of information. One of the innovative uses of nuclei segmentation has been as a means of preserving the spatial patterns of cells in digital pathology slides and thereby developing novel representation of images. Zheng et al. used a stacked network of CNNs for a hierarchical detection of nuclei, where nuclei detected in patches were grouped into blocks, and blocks ultimately grouped into images that retained granular nuclei patterns^6^. Training of this network was facilitated by use of an autoencoder to pretrain the network on unlabeled images. More generally, this use of self-supervised learning, where network architectures or loss functions enable networks to develop useful representation of images when labeled data may be unavailable or only partially available, has shown promise in developing representations for nuclei detection. Feng et al. similarly pretrained a stacked autoencoder with raw, unlabeled medical images, and found a classifier using this network was remarkably accurate in nuclei detection^7^. Combining insights from self-supervised learning and nuclei spatial content may hold exciting avenues for rapidly scaling sophisticated spatial analyses of images using DL methods.

More than simply recognizing nuclei, DL has also been used to extract second-order features of nuclei, such as chromatin texture, nuclear staining intensity and features of the nuclear envelope, and learn associations of such features with cancer genomics or recurrence. DL systems have found that nuclei tubule prominence, intensity, multicentricity, shape and texture, may predict OncotypeDx score^8,9^, long-term survival and pathologic complete response (pCR) in select cohorts of patients^10,11^. Features of nuclei have even been repurposed to produce measures of clinical grade, with AI predicted grade based on ‘nucleolar prominence’ potentially stratifying survival in stage 3 breast cancers more accurately than the more traditional Nottingham grade^12^.

A final application of nuclei detection and segmentation has been as a means of standardizing measurements of samples made by pathologists. Whilst it has been shown that measurements of cellularity could be standardized by classifying cell types based on nuclei^13^, one of the more distinctive applications of this was a study of Ki67, a measure of nuclear proliferation often limited in clinical use by lack of interpathologist consistency. In this study, an AI-empowered microscope was used to aid pathologists in assessing the Ki67 labeling index on 100 stained slides by applying algorithms to cells and providing these results to pathologists in real-time with augmented reality^14^. The study found consistency among pathologists substantially improved with the AI-enabled microscope compared to a conventional approach, particularly for more inexperienced operators.

##### Cellularity

Use of cell segmentation has also found applications in quantifying tumor cellularity (i.e. the surface area occupied by cancer cells or the ratio of cancer nuclei over the total number of nuclei in a histologic section). Historically done manually by pathologists, this process has been fraught with variability in cellularity measurements^15^. Over the years, numerous quantitative pipelines have been developed to assess cellularity. Most approaches have relied on DL to segment cellular areas in an image first and then either manually extract hand-crafted features or feed these ‘cellular’ regions to another machine-learning system, either another DL network or a more traditional classifier such as a support-vector machine^16,17^. Recent work has attempted to remove the hand-crafted feature engineering and instead develop end-to-end DL-systems that can directly assess cellularity from a WSI. Akhbar et al. was among the first to show the improved accuracy of end-to-end systems^18,19^. Rakhlin et al. directly compared different approaches, from using an initial segmentation to directly assessing cellularity, again showing the direct cellularity assessment with a DL model achieved a remarkably high Cohen’s kappa score of 0.69 (CI: 0.64–0.73) compared to the scoring provided by expert pathologists, with previous scores in the literature at 0.42^15^. Somewhat contrary to this, other studies have found that while the features extracted from DL systems are preferred, some additional processing, through dimensionality reduction may improve model accuracy^20^. Regardless, more work is needed to fully validate the optimal pipeline for cellularity measures. Perhaps more important for this area of research is improving model performance in borderline cases. An FDA review of numerous DL studies of cellularity assessment noted that, almost uniformly, such approaches misclassified the most challenging cases. Adenosis, a benign condition that at times mimics invasive breast cancer, was commonly labeled high cellularity by DL models and lobular carcinoma, known for its more distorted and non-cohesive architectures, often had cellularity underestimated^21^. It is precisely these challenging cases where a DL system would be of most benefit in aiding clinician judgment, suggesting that more work is needed to adapt models for real-world use (Fig. 3).

##### Microenvirenment and Stroma

Digital pathology is a lens through which we can examine individual cells, allowing the mobilization of spatial information for specific cell types into new ways to assess biology or treatment response. One exciting application of this has been the study of tumor-infiltrating lymphocytes (TILs). Stromal TILs have shown prognostic significance in triple-negative and HER2 + breast cancers^22^ and high density of TILs have promise in predicting pCR in HER2 + patients receiving neoadjuvant chemotherapy (NAC)^23^. Recent efforts have focused on DL methods to further extract TIL information from histopathology and uncover further biological insights.

Numerous studies have validated the effectiveness of DL pipelines in reliably recognizing both TILs and their distribution in WSIs. In specimens stained with an antibody to CD45 (also known as leukocyte common antigen), a receptor tyrosine phosphatase expressed on leukocytes, immune rich regions containing TILs could readily be distinguished from immune poor regions, with an F-score (a measure of accuracy that balances both precision and recall) of 0.94 compared to pathologist-derived F-scores of 0.88^24^. More direct estimation methods, without the use of specialized stains, have relied on networks that segment or recognize individual cells based on morphology. In one of the more impressive examples of this, Swiderska et al. first used the well-known YOLO (You Only Look Once) algorithm, an efficient method for object localization, to obtain bounding boxes for TILs in a given slide and then within such boxes, segment the cell body and membranes of lymphocytes^25^. This network identified specific CD3 and CD8 positive lymphocytes, with an F-score of 0.82. While impressive, the effort required extraordinary manual labor, as ~170,000 lymphocytes had to be labeled by trained pathologists on slides for use in the model. Other efforts for TIL recognition have used the approach of first detecting nuclei and then classifying cell types as lymphocytes^26–28^.

Beyond simple recognition of TILs, more recent efforts have used DL to integrate the spatial distribution of TILs with novel predictions on tumor behavior, genomic alterations^29^, responses to PD-1 checkpoint inhibition therapy^30^ and prognosis^31^. Narayanan et al. studied TIL localization in the context of ductal carcinoma in-situ (DCIS). To do this, the authors used a modified U-NET architecture to detect DCIS areas in a slide. A cell detector was used to segment cells in this area with a CNN-based classifier ultimately categorizing these cells into epithelial, stromal or lymphocytes. A ‘colocalization’ formula applied to these regions provided a quantitative measure of the degree of TIL clustering in regions of interest. The approach showed that TIL colocalization varied with the presence of invasive carcinoma; whilst sections in which DCIS was found next to cancer had lower numbers of TILs overall, they had much higher degree of colocalization of TILs around DCIS ducts, lending support for a more immune reactive microenvironment in disease states with worse prognosis^32^. In another study, Lu et al. also used a U-NET to detect individual lymphocytes, although counts of lymphocytes were aggregated into TIL scores per WSI. This aggregated score correlated with expression of specific genes in the immune response pathway (such as *CTLA4*) and interestingly, such genes seemed to differ across breast cancer subtypes^33^. Our understanding of the spatial arrangement of TILs continues to expand, as recently even the arrangement of TILs with particular shapes, circular or elongated^30^, has shown to have therapeutic insight into breast cancer^30^. Attempts to integrate TIL assessment into a clinical workflow are already underway in clinical trials^22^. DL systems will streamline this process and, more importantly, continue to aid in uncovering morphometric and spatial cellular patterns that reflect a variety of biologic and therapeutic insights that may advance clinical practice.

Whilst historically the stroma in WSIs has received relatively little attention compared to epithelium as a source of useful information about tumors, increasingly this is changing^34^. Biologically, new insights are emerging on the role of the stroma in enabling tumor growth and metastases through cytokine or chemokine-mediated secretion^35^. Additionally, abnormalities in the stroma are often quite subtle and difficult to distinguish by eye, with emerging data suggesting that pattern recognition systems are better suited for identifying these changes^36^. As a whole, DL offers a potential means to characterize and perhaps utilize yet to be explored stromal information.

Numerous studies have already validated the use of DL networks to segment stroma effectively automatically from epithelia^34^. Biomarkers derived from stroma by such networks, including tumor-stromal ratio^37,38^ and even collagen orientation^39,40^, have been associated with overall survival in breast cancer and DCIS recurrence after surgery^41,42^. The rich information content hidden in the stroma of tissue slides is perhaps best highlighted by Bejnordi et al., where three CNN networks were trained in hierarchical fashion to segment tissue slide stroma, identify tumor-stromal content and then develop a score for overall likelihood of tumor malignancy^36^. Surprisingly, in subsequently examining the network’s behavior, the authors found that higher grade DCIS had a higher mean stromal content within a certain distance of DCIS margins compared to lower grade cases. In this way, the network could act as a stratifier of DCIS without even being trained to recognize DCIS. Hence, stromal content will likely increasingly be an area ripe for new biomarker discovery and novel insights into tumor biology^43^.

Tumor-associated collagen is increasingly garnering attention as an example of DL-derived, entirely novel microenvironment biomarker. From facilitating movement of malignant cells and therefore metastasis to biomechanically triggering signaling pathways, collagen is thought to have a variety of roles in tumor behavior. Motivated by this, one study^39^ used DL to segment collagen tissue from bright field histology images. Using 37 extracted features relating to orientation, texture, and alignment, the authors were able to stratify hormone receptor positive invasive ductal carcinoma into prognostic groups, with specific factors including orientation magnitude and fiber width most significant^39^. In an extension of this, Li et al. developed a quantifiable measure of collagen disorder^40^. With a conditional general adversarial network, a DL approach using competing objectives to train a ‘generator’ and ‘discriminator’, the authors separated epithelial and stromal tissue and subsequently used a derivative of a gaussian model to classify the orientation of collagen in the stroma, particularly at the tumor border. Entropy-derived quantification of collagen disorder was then used to estimate prognosis, with interestingly more ‘ordered’ frameworks associating with significantly lower 5-year disease-free survival^40^.

### Clinical characteristics

One of the most long-standing yet significant clinical challenges in breast cancer remains estimating likelihood of progression or response to therapy, especially in early stages^1^. Some of these challenges are highlighted by the low response rate, estimated at 4–25%, of patients to the top 10 most highly prescribed US drugs^1^. AI, specifically DL, can substantially aid in therapy decisions by maximizing the extraction of clinical information from multi-modal sources^44,45^ as well as in uncovering response biomarkers perhaps less obvious to the human eye, and, as a result, has been at the center of recent FDA initiatives to personalize therapy decisions^1^. In Europe, the CE mark has already been given to the breast cancer detection algorithms developed by Paige.AI, which helps pathologists in the detection of specific foci in slides suspicious for cancer and Ibex’s Galen, which has demonstrated high accuracy in distinguishing subtypes of invasive breast cancer and grading DCIS. Beyond detection of breast cancer, early studies have validated that incorporation of signals from digital pathology can also aid in capturing treatment responses in ER- patients^46^.

In no area of breast cancer is recurrence estimation both as significant and vexing as it is with ER+ cancers. Numerous genotype tests, including the Oncotype DX, have been developed to aid in predicting which early-stage patients may benefit from more aggressive initial treatment^47^. DL efforts have attempted to identify tissue correlates that accurately reflect the OncoDX score and thus make these results more widely available for clinical use. In one such study, a DL pipeline was used to first segment nuclei and then separate epithelial and stromal tissues prior to extracting 216 nuclei features relating to shape, architecture, and orientation^47^. These direct nuclear features, thought to better capture the nuclear distortions reflected by clinical grade, were found to predict high and low Oncotype Dx scores. More interestingly, predictive performance improved when the model was initially trained by grouping intermediate with low Oncotype scores, suggesting that histomorphometrically these groups may be quite similar. Other studies have used DL to extract nuclei features such as the ratio of tubule nuclei^9^ or number of mitotic events^8^ to again obtain surrogates for grade that might be used to directly predict Oncotype Dx categories. Albeit good in distinguishing extreme cases, with the best AUC of 0.83^8,9^, these methods have thus far proven only mildly effective at assessing risk levels in more indeterminate cases, with either borderline risk scores or conflicting clinical grade. While not specifically DL, some studies have shown promise in extracting novel biomarkers, such as heterogeneity of nuclei polarity^11^ or immune cell clustering^31^ across a tissue sample, to directly estimate survival in ER + patients. Additional studies have incorporated histopathology features extracted from DL systems with clinical features, including Magee features^48^ or common clinical characteristics incorporated into clinical nomograms^49^. Howard et. al., who combined tile-derived recurrence prediction scores with clinically-derived scores, interestingly did not exclude intermediate risk patients and reliably detected low-risk subgroups of patients that would not benefit from further genomic testing. Taken together, novel biomarkers and multi-modal data integration may hold promise in improving estimates of recurrence for ER + patients, particularly in more ‘ambiguous’ clinical scenarios.

Recent work has also explored the use of DL to predict pCR. Li et al. employed networks trained directly from WSIs in patients receiving neoadjuvant chemotherapy to produce a pCR ‘score’. This approach surprisingly achieved an AUC of 0.84^50^ in predicting pCR, and was unique in that no manual pre-processing, that specified a-priori and extracted cell or nuclei morphologic features, was done as in many of the other studies examined^51^. Of note, the pCR performance was across patients with a mix of hormone receptor status and notably triple-negative breast cancer patients trended to non-significant pCR score distribution compared to other subtypes. This difficulty in predicting neoadjuvant therapy response in triple-negative patients was echoed by Naylor et. al. in a study of 350 slides^52^. The study used the familiar approach of encoding tiles of a slide using pre-trained networks and then aggregating encodings using either average or attention pooling methods, finding the most successful combination of modules yielded a low AUC of 0.64^52^. Other work in HER2 and triple-negative breast cancer patients utilized separate models for tumor detection and nuclei segmentation, with subsequently extracted nuclear features used to predict pCR, finding nuclear intensity and texture-based features most reliably predicted pCR^10^. To date the most promising results in triple-negative disease have come from Duanmu et. al., in which a novel ‘knowledge-derived’ spatial attention mechanism was employed to focus a DL system on tumor areas with highest information content for its prediction task^53^. To do so, serial slides, including H&E, Ki67 and Phosphohistone H3 (PHH3) were used, with the latter two obtained by immunohistochemical analysis. A DL network was used first to identify and segment tumor cells on H&E slides. For the corresponding sample, Ki67 and PHH3 areas were identified and based on these biomarker-positive areas, an attention map was generated to more highly weight tumor cells contained within this spatial region of interest. Images containing the resulting cells were then fed through a more typical Resnet-34 to obtain predicted pCR. The performance of the resulting system is quite impressive, with AUC’s on a tile basis of 0.96.

Direct survival estimation from tissue pathology remains a challenge^54,55^. There is evidence to suggest that the combination of both cell and micro-environment level features, such as stromal content, along with clinical variables improves predictions from cell-level features alone^56^. This is consistent with the notion that DL networks with the capacity to attend to multiple levels of a hierarchical representation of an input may be an avenue to learn more meaningful relationships related to survival prediction.

### Molecular features

Pipelines for robustly extracting genomic information from WSI solely or as part of combinatorial biomarkers hold the potential to change a variety of clinical practices, from predicting responses to therapy to improving staging or discovering novel biomarkers^2,57–60^. Numerous studies have already established that the morphologic traits of tumor cells, extracted by pathologists themselves, are associated with expression of specific genes^60–62^. Recent work applying DL techniques with histopathology has extended this insight to an unprecedented level, allowing a spatial and morphologic mapping of cancers to genetic, proteomic, and expression data.

Spatial transcriptomics directly maps expression data to cells in histopathology images. Due to the expense and more limited availability of these techniques, few datasets have been available to integrate this technology with DL in studying breast cancer. In one such study, He et al. developed ST-net, a DL algorithm to capture gene expression heterogeneity of 250 genes^63^. Like many DL approaches, WSI were first broken into smaller ‘tiles’ of image size 250 × 250 pixels, from which expression data of the 250 genes was directly trained on and predicted. The authors found 102 genes could be successfully predicted based on histopathology alone, and these results correlated well with RNA seq data from a comparison set of data used from TCGA. More impressively, the method demonstrated the feasibility of this approach in discovering novel genes involved in breast cancer pathways, with the authors discovering three genes that were markers of tumor growth and immune activation, respectively^63^.

Other studies have used more indirect approaches to relate the spatial content of WSIs to genomic mutations. In evaluating point mutations in breast cancer, Qu et al. achieved a high Area Under the Curve (AUC) in predicting point mutations in *TP53*, *RB1*, *CDH1*, *NF1* and *NOTCH2* and copy number alterations (CNA) in 6 additional genes^64^. The pipeline employed a classic convolutional neural network (i.e. Resnet) as the backbone to ‘encode’ tile image features and combined these using an attention-mechanism to make whole-slide level predictions of mutation status. Other approaches have leveraged larger sources of data across a range of cancer types to make genomic predictions. In one such study, Kather et al. used an exceedingly computationally efficient convolution network, Shufflenet, to extract image features across thousands of colon, lung, breast and a range of other cancer images, and used these features to predict both point mutations and molecular pathways. In breast cancer, this approach reliably detected both *TP53* and *PIK3CA* genomic alterations^65^. In another large-scale, pan-cancer approach, 17,000 hematoxylin and eosin stain (H&E) slides were used train a DL network to correlate computational histopathologic features to recurrent genetic abnormalities across cancers, including whole genome duplications, focal amplifications and deletions and driver gene mutations. The study impressively demonstrated that across virtually all cancer-types, including breast cancer, tumor morphology can reliably be shown to be associated with genomic alterations^66^. These pan-cancer studies have important limitations, however, given that several mutations known to predict a cancer phenotype could not be detected. *CDH1* mutations, for instance, which, while almost pathognomonic of invasive lobular carcinoma, could not be predicted in Kather et al. from the H&E images. Thus, although these studies have undoubtedly proven the principle of relating histologic phenotypes to genomic markers, much work remains on reliably extracting this information in a predictable manner in a routinely implementable system.

DL has similarly been employed to reliably distinguish molecular subtypes of breast cancer from histopathology and more impressively, capture molecular heterogeneity reflected in a WSI without the expression data at a cellular level that spatial transcriptomics provides^65,67^. Jaber et al. combined a DL feature extractor with a support vector machine classifier to categorize tiles of a WSI into PAM50 subtypes. While slide level predictions were aggregated by a ‘voting mechanism’ across tiles, the tile-level predictions themselves reflected a ‘heterogeneity’ of subtypes present in the tumor sample. Interestingly, the degree of this heterogeneity had prognostic significance, as patients with overall Luminal A subtype breast cancer but with basal subclones had poorer survival compared to homogenous Luminal A tumors^67^. These results demonstrate that DL approaches can provide novel insights into heterogeneity that may warrant more detailed molecular and clinical response analysis. Extending this concept further, newly designed DL approaches, such as ‘discriminative bag of cells’^68^, have used cell and nuclei features to define novel cell types present in a tumor sample. Histograms of these cell types can then be used directly to predict molecular subtypes present in breast tumor samples, more explicitly ‘quantifying’ the cell morphologic features that have unique signatures across subtypes. In this way, 8 unique cell types have been defined across the basal molecular subtype.

Methods to infer trait expression or perform gene enrichment analysis, through prediction of RNA expression from histopathology, have also been undertaken. In the first study linking transcriptome-wide expression to morphology in breast cancer, Wang et al. studied over 17,000 genes and found that convolutional networks could reliably predict mRNA expression, validated through RNA sequencing, of approximately 9000 genes^69^. The scale of the analysis was particularly impressive, with each gene requiring an independent model that could predict RNA seq data at the tile level. Based on mRNA expression estimates, the authors isolated 16 enriched gene pathways, most of these known to be involved in breast cancer pathogenesis. Moreover, the spatial variability of expression, by using the tile-level predictions, was significantly associated with spatial transcriptomics profiling in 59 of 76 genes^69^, showing the validity of such approaches in providing spatially relevant, genomic insight^70,71^. Similar tile-level DL systems have been used to create ‘heterogeneity maps’ in mRNA expression that have prognostic significance^70^, define traits such as *MKI67* and *FOXA1*^70^, and show ‘signatures’ that distinctly mark each of the breast cancer molecular subtypes^71,72^. Spatial mapping of expression data has also been used to help define novel biologic differences in tissue or cell type. Using DL to segment stroma and epithelium and then training a classifier using tissue type to predict mRNA expression, has revealed that genes correlated with expression vary not only between tissue type but across molecular subtypes of breast cancer. For instance, while epithelial tissues were enriched in genes related to processes such as cell cycle, estrogen-receptor (ER) positive subtype breast cancers specifically had enriched G1 and G2 phase genes, while triple-negative cancers were enriched for mitotic phase expressed genes^38^. This concept of mapping expression data to imaging cell type has been extended to reliably predict populations of T and B cells in WSI^66,71^.

Hormone receptor status similarly reflects protein expression, and DL systems have reliability estimated such status, compared to immunohistochemistry, from morphologic characteristics of cells on pathology slides alone. While pan cancer approaches have been able to accurately assess ER status^65^, the most successful applications have come from studies solely on breast cancer cohorts. In one such study, a simple Resnet architecture used to encode tile level features combined with a slide level L1 logistic regression achieved PPVs of 98%, NPVs of 76%, and accuracy 92%, noninferior to traditional immunohistochemistry in predicting ER status (PPV, 91–98%; NPV, 51–78%; and accuracy, 81–90%)^73^. Perhaps surprisingly, these morphologic features relating to receptor status, can be learned without having paired labels (i.e. receptor status) with training data (i.e. WSIs). In a self-supervised learning approach, Rawat et al. learned histologic features for WSIs ‘automatically’ by splitting images into halves and by having a system predict which two halves belong together^74^. Using groups of similar features learned this way resulted in a better classifier compared to directly using image patches for ER, with AUCs of 0.88 vs. 0.82, respectively, suggesting that the features were separated not only on morphological but also biologic information learned by the network. This exciting finding may pave the way for pre-training a variety of networks which can then be used to automate receptor status identification. To capture the relevant histologic features more directly and use these in predicting receptor status, Naik et al. used an attention mechanism as part of multiple-instance learning^75^. In this system, bags of tiles from each slide are given weights, learned by the DL model, for use in predicting slide level characteristics, such as ER status. This approach yielded amongst the highest AUC, 0.92, reported for ER receptor prediction. Further, the findings correlated with known biology. ER+ tiles for instance showed two distinct types of features: with uniform cells with small nuclei and low mitotic rates consistent with low grade tumors; or linear arrays surrounded by stroma with no duct formation consistent with invasive lobular carcinoma-type. MIL methods have still proven effective when ER+ and ER- groups are balanced with similar numbers of low/intermediate grade samples^76^.

Predicting HER2 status from WSIs has in large part been less accurate than for ER receptor status, with the best reported model AUC’s around 0.82^77–79^. An interesting direction for this area of research, however, has been model estimation of HER2 status as a more reliable measure of response to trastuzumab therapy. Bichcky et al. trained a relatively simple DL network to directly predict gene-amplification status in a cohort of patients who subsequently went on to receive trastuzumab. Surprisingly, patients with gene amplification did worse with therapy if they had low model-predicted HER2 score and better if their model-predicted scores were higher^80^. In this way, inferring at a tile level HER2 status and aggregating this at a slide level in an intelligent manner may be a more accurate means of estimating the receptor expression phenotype in a sample overall. This has direct therapeutic relevance, especially considering the recent DESTINY-Breast04, in which a new category of breast cancer patients, HER2-low, had PFS and OS meaningfully improved by use of trastuzumab deruxtecan (TDxd)^81^, leading to FDA approval for use of TDxd for this indication. Using AI-based methods to quantify and then stratify HER2 expression phenotypes more accurately could thereby, very directly, accelerate the adoption of an entirely new approach to breast cancer treatment.

Finally, estimation of DNA repair deficiency from histopathology, an area with tremendous therapeutic potential, while in its infancy, has been explored in a few reports. DL Resnet architectures have successfully classified tumors as having high or low fractional genome instability (or CIN), a measure which was shown to correlate with survival^82^. More directly, other work has used slide imaging to directly infer homologous repair deficiency (HRD) via *BRCA* mutation status^83^ and LST signatures^84^. Lazard et. al. expanded on this by developing a novel visualization approach that applies the slide-level attention weighting to individual tiles, thereby estimating a probability for each tile in a slide having HRD. Using this tool, the authors found morphologic correlates of HRD status in slides, some well-described in the literature such as necrosis and high density of TILs, whereas others less-discussed, such as intratumoral fibrosis and abundant carcinomatosis with clear cytoplasm^84^. The DeepSmile system enhanced performance by incorporating self-supervised learning, namely SimCLR, to pretrain a network to learn useful feature representations of tiles and then feed these representations to a multiple instance learning network to ultimately predict HRD status, achieving an impressive AUC of 0.84 on the TCGA dataset^85^. Overall, readily recognizing morphologic correlates of HRD status can have direct therapeutic relevance particularly if such findings can be extended to infer the more general “BRCAness” phenotype with implied sensitivity to PARP inhibitors^60,86^.

### Challenges and outlook of AI in breast pathology

How exactly DL systems will be utilized in clinical care is evolving in a fascinating manner. Yet despite the exciting technological and scientific advances in the field and its applications in healthcare, numerous challenges remain to realizing its implementation in routine clinical care.

Perhaps most fundamental are the cultural and institutional attitudes towards the acceptance of such systems in standard practice. Akin to the great microscopy debate in the Paris Academy of Medicine in the nineteenth century^87^, physician attitudes towards use of AI in practice are often mired with skepticism and preconceived notions of the ‘right’ roles for AI in care. In surveys, this physician sentiment on the division of roles between themselves and DL-systems, often comes not from a comprehensive review of where clinical care might be augmented by DL-systems (error rate reduction, integrating multimodal data, or extracting new types of biologic insights), but rather fixed notions of what parts of a clinical workflow should be shielded from incursion^88^. Integrating AI into sustainable clinical workflows is an entire paradigm shift in care and only by reimagining current practice can the full potential of any such systems emerge.

Another challenge in AI uptake in healthcare is the blackbox nature of how these systems, especially weakly-supervised DL systems, arrive at their predictions. Although the relationship between inputs and final predictions of DL networks cannot be easily mapped to a handful of variables and the semantics cannot be trivially applied to the features utilized for a given label prediction, much can still be gleaned from how networks translate inputs to outputs. Two relatively recent approaches we discuss here include use of attention weights and knowledge distillation. The key behind the attention mechanism is that inputs are weighted by their relevance in determining the final output. In the medical domain, sorting attention weights of positions in a sequence of inputs to a DL network has allowed researchers to highlight the most relevant times for critical events in an ICU or sites in a genome where HIV is likely to integrate^89^. With imaging, as already discussed, attention weights can be used to create maps of tiles in a slide with features most salient in arriving at the slide class label. This can focus a pathologist’s eyes to the tiles with morphologic characteristics of greatest interest. Knowledge-distillation, another emerging technique, involves taking a more complicated network and training a simpler, student, network to arrive at the same predictions. This simpler network, more interpretable, has been used to develop rule-extraction networks for complicated prediction tasks such as ICU mortality prediction or diagnosing Alzheimer’s disease^89^. Explainable AI will continue to require dedicated research effort that not only illuminates how networks process inputs but also how this can then be distilled to key pieces of information that can augment clinical practice.

Building off the ‘black-box’ nature of DL systems, a third challenge is ensuring the findings from systems applied to specific data types are appropriately generalizable. In general, models aimed at predicting low incident events can have inflated performance, especially if such models make indiscriminate predictions. This is especially relevant for genetic alterations which can often be low frequency events in datasets. Appropriately assessing model predictions, via combining precision/recall analysis with more general definitions of ‘accuracy’ can help tease out the true predictions a model is making and allow more fine-tuning of performance. A more specific issue in the context of the DL is when such systems infer outcomes from unintended aspects of how the data generated. In an analysis of TCGA digital histologic images, Howard et. al. found that staining differences between submitting sites can be detected by DL systems, despite generally used color normalization methods and, more worrisome, such differences lead to biased predictions^90^. The study highlights the specific need to ensure DL models, capable of learning a multitude of complex relationships between data inputs, learn only on the ‘appropriate’ aspects of datasets. It should also be noted that in multiple studies, rather than developing algorithms based on TCGA WSIs, investigators utilized this publicly available dataset as the essential external validation dataset^91^ for the validation of algorithms developed through the analysis of purpose-built WSI collections. Given the potential sources of noise in the TCGA WSI dataset, caution should be exercised in the assessment of the performance of TCGA-derived or TCGA-validated AI algorithms.

A third challenge in DL adoption in pathology is a general requirement of these systems: the need for ground truth (i.e. labeled data). This is particularly relevant in the context of strongly supervised approaches, where annotating data is a time and labor-intensive process. In healthcare this is compounded by data being held in disparate silos in non-uniform formatting, requiring an inordinate amount of effort to collect and label such samples. One approach to deal with this is to make the labeling process ‘easier’. A creative initiative being explored is the use of Amazon Mechanical Turk and other platforms to crowdsource annotations^92^. This work has even been extended such that medical images can be represented on a video game ‘canvas’ to help popularize annotation tasks^92^. Another approach to the labeling problem is to leverage self-supervised or even unsupervised learning to drastically reduce the amount of labeled data needed to make meaningful inferences (see Box 2 for definitions). Self-supervised techniques allow networks to learn representations of data without explicit labels by employing contrastive techniques - because no labels are needed, huge amounts of data may be fed to a network to ‘prime’ it. A smaller amount of labeled data may then be used to ‘fine-tune’ the performance of the network and in this way only a small amount of truly labeled data is required. Graphical temporal networks^93^, manifold preserving autoencoders^7^ and tensor decomposition^94^ are but some specific examples of this approach that have been explored in breast cancer.

A fourth barrier is the specificity of domain knowledge in healthcare. Having networks that a priori can mimic the type of filtering done by medical practitioners is an open area of exploration^95^. Leveraging past cases, through retrieval of images with comparable features^96,97^, allows for new approaches to diagnosis such as artificial voting and consensus building. Finally, and perhaps most ambitiously, use of multi-modal data, through combinations of mammography, histology and other clinical data will open new ways features of an individual’s specific disease state to clinically relevant outcomes^94,97,98^. Chen et al. found a multimodal fusion deep learning network that combined both molecular and histology based features outperformed models trained on only single modalities for survival prediction in breast cancer^99^. This study also highlighted the fact that the prognostic and/or predictive information that can be derived solely from the AI analysis of H&E WSIs may not suffice for the accurate prediction of outcomes of breast cancer patients, and that integrative models^100^ should be entertained.

A final challenge to adoption of DL networks in clinical pathology is more abstract yet vital: demonstrating improvements to existing workflows. Adoption of such systems requires that new workflows drastically, not incrementally, improve on old ones. Whilst a common focus in digital pathology has been on error reduction or standardization that AI approaches can provide in diagnostics, this is only the tip of the iceberg of how DL-systems might augment clinical capabilities. For instance, DL networks have been piloted in operating rooms, where virtual H&E staining performed on breast tissue samples via general adversarial networks allows a DL system to then analyze the tissue specimens in real-time to make diagnostic predictions^101^. It is only a marginal extension of this to imagine a system that detects margin-free resections or more aggressive pathologic features that may warrant real-time, intra-operative therapy. Beyond virtual image processing^102^, future clinical workflows that incorporate DL networks will likely extend to models that work with patients’ devices themselves^103^. DL systems integrated on patients’ devices have provided a real-time image recognition and analysis system for cosmetic results after breast cancer mastectomy and reconstruction^103^. The feedback and objective measures of outcomes will advance the capabilities of care to even greater precision. Continued adoption will require focused attention on how such clinical workflows be developed that benefit all stakeholders and augment current clinical capabilities. Together, many of the above innovations alongside the advancements in biologic, prognostic and therapeutic information extracted from WSIs themselves, hold the key to an array of remarkable pipelines that may change the way in which breast cancer care is delivered.

### Reporting summary

Further information on research design is available in the Nature Research Reporting Summary linked to this article.

## Supplementary information

Reporting Summary
